# Protected area designation and management in a world of climate change: A review of recommendations

**DOI:** 10.1007/s13280-022-01779-z

**Published:** 2022-08-23

**Authors:** Thomas Ranius, Lina A. Widenfalk, Meelis Seedre, Ly Lindman, Adam Felton, Aino Hämäläinen, Anna Filyushkina, Erik Öckinger

**Affiliations:** 1 Department of Ecology, Swedish University of Agricultural Sciences, Box 7044, 750 07 Uppsala, Sweden; 2 Greensway AB, Ulls väg 24A, 75651 Uppsala, Sweden; 3 Southern Swedish Forest Research Centre, Swedish University of Agricultural Sciences, Box 49, 230 53 Alnarp, Sweden; 4 Forest Department, Ministry of the Environment of Estonia, Narva mnt 7a, 15172 Tallinn, Estonia

**Keywords:** Climate change adaptation, Conservation, Migration, Spatial planning

## Abstract

Climate change is challenging conservation strategies for protected areas. To summarise current guidance, we systematically compiled recommendations from reviews of scientific literature (74 reviews fitting inclusion criteria) about how to adapt conservation strategies in the face of climate change. We focussed on strategies for designation and management of protected areas in terrestrial landscapes, in boreal and temperate regions. Most recommendations belonged to one of five dominating categories: (i) Ensure sufficient connectivity; (ii) Protect climate refugia; (iii) Protect a few large rather than many small areas; (iv) Protect areas predicted to become important for biodiversity in the future; and (v) Complement permanently protected areas with temporary protection. The uncertainties and risks caused by climate change imply that additional conservation efforts are necessary to reach conservation goals. To protect biodiversity in the future, traditional biodiversity conservation strategies should be combined with strategies purposely developed in response to a warming climate.

**Supplementary Information:**

The online version contains supplementary material available at 10.1007/s13280-022-01779-z.

## Introduction

Biodiversity conservation usually focuses on maintaining the current, or restoring the historical, status of biodiversity. Areas can be protected to provide refugia for species from overharvesting, habitat loss and habitat degradation. Climate change is an increasing threat to biodiversity, and even within the permeable borders of protected areas, climate change readily makes conditions unsuitable for many species (Monzón et al. 2011). Thus, climate change challenges which species can persist in a given protected area, and how best to prioritize among sites and habitat types which to protect (Bellard et al. 2013). Consequently, one of the great challenges for today’s conservation managers is to adapt strategies for biodiversity conservation to the consequences of ongoing climate change.

**Fig. 1:**
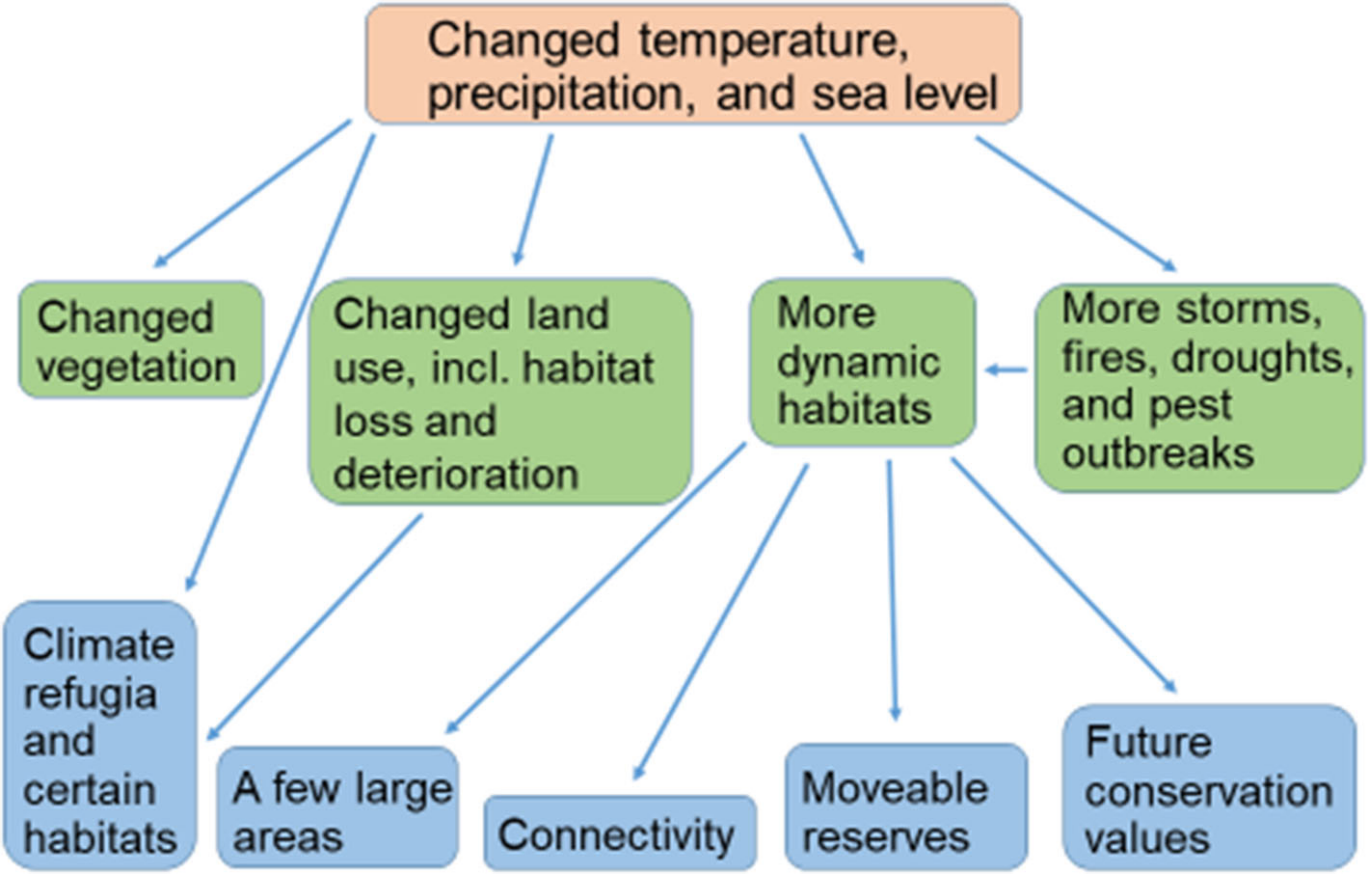
Direct (red) and indirect (green) effects of climate change, and how they lend main arguments to the five most common types of recommendations for future area protection that we found in the scientific literature (blue)

Climate change affects biodiversity via multiple direct and indirect pathways (Fig. 1). Altered temperature and precipitation patterns, and higher sea levels are direct effects of climate change (IPCC 2014). In addition, climate change can induce indirect changes to environmental conditions for biodiversity, via both natural and human-induced processes. Natural processes include changes to disturbance regimes involving fires, storms, floods and droughts (e.g. Foster 2001). Moreover, altered growing conditions can change vegetation characteristics, for instance in terms of increased vegetation height and productivity (Elmendorf et al. 2012) or lost natural canopy cover (Martin et al. 2015). Other indirect effects stem from human responses to climate change, especially those involving climate change adaptation and mitigation strategies in land management (Lindenmayer et al. 2012). These include an increased extent and intensity of forestry and agriculture, changes to the composition of crops and trees grown, and additional modifications to the specifics of management regimes (Delcour et al. 2015; Felton et al. 2016).

Both the direct and indirect effects of climate change raise significant questions about the continued effectiveness of biodiversity conservation strategies involving protected areas. Here we synthesize relevant knowledge in this regard, by addressing the question of how to adapt the use of protected areas to effectively conserve biodiversity despite the projected impacts of climate change. To do so we synthesized recommendations provided by scientific review papers involving designation and management of protected areas in forest and agricultural landscapes within the temperate and boreal zone. Most previous recommendations and reviews are limited to the direct effects (e.g. Heller and Zavaleta 2009; McLaughlin et al. 2022). In contrast, here we considered both direct and indirect effects of climate change, since conservation managers typically have to account for both types of effects at the same time. Our main questions were:

1. Which conservation strategies for designation and management of protected areas are recommended to improve environmental conditions for biodiversity, given the effects of climate change? To understand under which circumstances recommendations are relevant, their links to ecological processes are important. Therefore, in the Discussion, we link the arguments behind the recommendations to ecological theories, even though that was only done in some of the papers we synthesized.
2. To assess the generality of these recommendations, we also asked: Do recommendations vary depending on whether they are primarily in response to the direct or indirect effects of climate change and which aspects of biodiversity are intended to be protected (e.g. overall biodiversity, threatened species, or ecosystem services)?

## Methods

We systematically searched for scientific review articles addressing areas protected for biodiversity conservation in forest and agricultural landscapes in the boreal and temperate biomes of the Northern hemisphere (Appendix S1). We considered only reviews, since we were interested in recommendations for conservation strategies rather than in the results from individual case studies. Review papers typically base their recommendations on multiple cases, and therefore, are able to draw more general recommendations than original research papers. Our geographical focus was linked to the higher projected climate warming at high latitudes of the Northern hemisphere (IPCC 2014), which increases the importance of climate warming as a consideration in conservation strategies. We searched for recommendations regarding the designations and management of protected areas that were assigned greater relevance due to current and projected climate change. We defined the likely direct and indirect effects of climate change that may affect conditions for biodiversity by discussions and reading literature (Table 1).

**Table 1:** Direct and indirect effects of climate change that might affect biodiversity, considered in this study

Effects	References
**DIRECT EFFECTS**	
Increased temperature	IPCC (2014)
Changed levels of precipitation and snow cover	IPCC (2014)
Rise of sea level	IPCC (2014), Mengel et al. (2016)
Unspecified climate change	IPCC (2014)
**INDIRECT EFFECTS**	
**Disturbances and catastrophes**	
More flooding, drought, storms, and fires	IPCC (2014) and Seidl et al. (2017)
**Habitat loss and changes**	
Changes in the amount of agricultural land	Ramankutty et al. (2002)
Changes in the amount of forested land	Scheffer et al. (2012)
Increase in exotic tree species	Felton et al. (2016)
Increase in native broadleaved trees	Felton et al. (2016)
Changed vegetation composition	Elmendorf et al. (2012) and Martin et al. (2019)
Shorter rotations in forestry	Roberge et al. (2016)
Longer season of vegetation growth and grazing	Garonna et al. (2016)
** Pests and invasive species**	
More invasive species	Bellard et al. (2013)
More pest and pathogens	Sturrock et al. (2011) and Seidl et al. (2017)
Higher use of pesticides	Delcour et al. (2015)

**Fig. 2:**
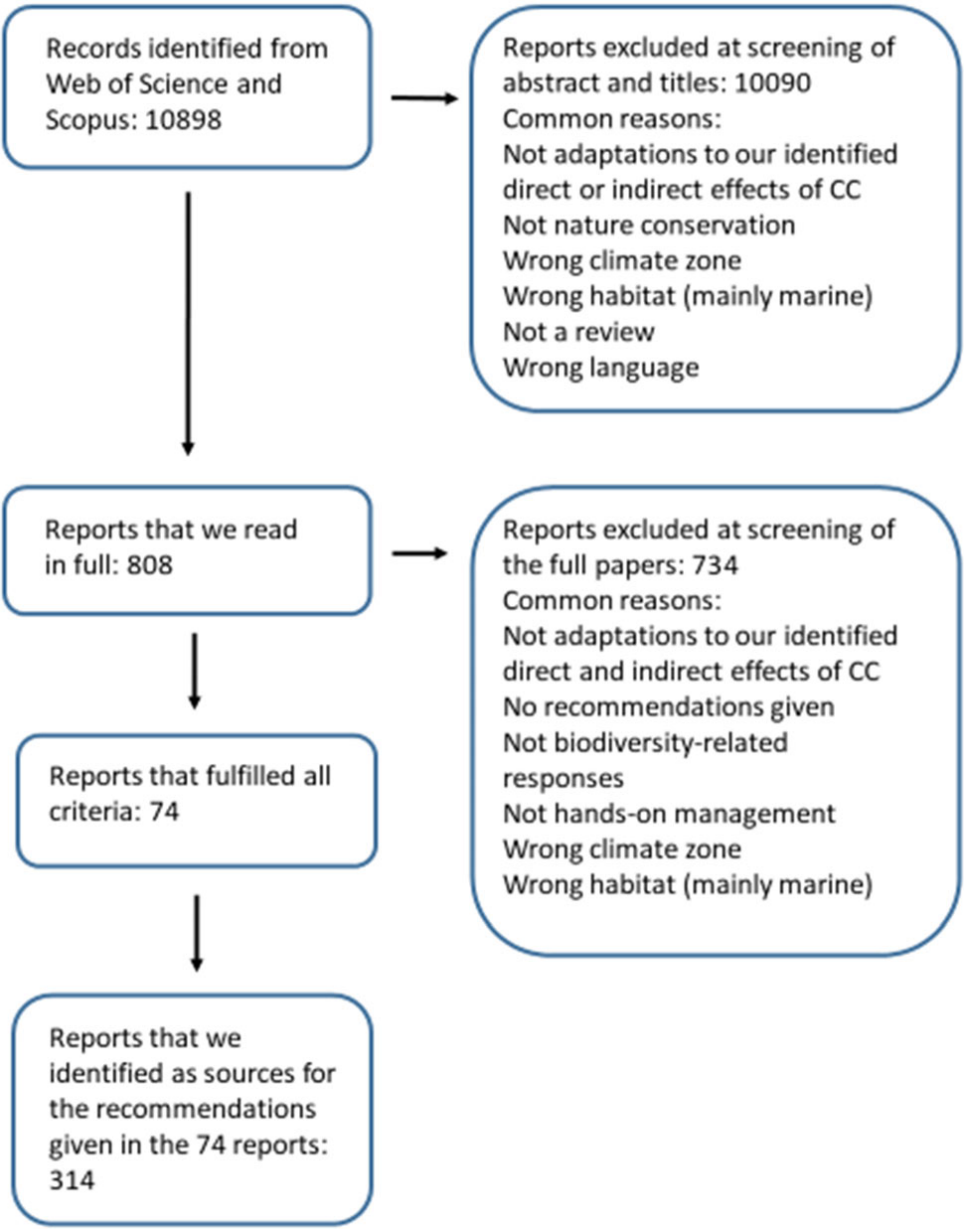
Our search for relevant literature: numbers of reports excluded and retained at different steps

The search was first conducted on September 13th 2018 and updated on November 10th 2021 in two literature databases: Web of Science Core Collection and Scopus, combining three search strings (included in all searches) with five other search strings (included one at a time; Appendix S1). From these searches, we obtained in total 10 898 references (Fig. 2). We screened all articles for relevance, first at title and abstract level, and then at full text level (808 articles), using predefined inclusion criteria (see below). The applicability of the inclusion criteria was tested by comparing agreement across two members of the project group at abstract level screening using a subset of 50 abstracts. Disagreements were discussed and the inclusion criteria refined where necessary. Level of agreement was assessed by Fleiss’ kappa test (Fleiss et al. 2003). If the agreement score was below 0.6, a further 50 abstracts were screened following discussion and further refinement of the inclusion criteria. This process was repeated three times, until a score > 0.6 was reached, resulting in the final version presented in Appendix S2.

For all papers included in the final selection (74 reviews), we categorized their recommendations using a four level hierarchical classification. The three highest levels are shown in Table 2. The fourth were the most detailed level (Table S1). For recommendations that were more generally formulated, this level was not used. The categories were partly defined before the classification started, and partly adapted to the recommendations found in the papers, since we added and modified categories during the classification. Recommendations should consider ‘hands-on’ conservation measures (and thus, for instance, not only about monitoring, research, governance, or information activities), and be presented as conclusions from the reviews (i.e. typically in the Discussion section). We recorded the original research papers (which could be one or more papers, but in some cases reference to an original paper was lacking) that were referred to as a support for the recommendations (Table S1). For each class of recommendations, we counted the total number of referred original papers that were provided in support. To avoid counting any original papers more than once, we cross-checked for redundancy, since many original papers were cited in several review papers. This was done separately for each of the direct and indirect effects (Table 1), and for different aspects of biodiversity (i.e., diversity of species, genes or habitats, certain threatened species, and ecosystem services dependent on biodiversity). Since a recent review found only small changes in the recommendations over time (McLaughlin et al. 2022), we did not separate recommendations from different time periods.

**Table 2:** The number of original papers providing statements supportive of particular categories of recommendation in the 74 reviews. Recommendations given here are the categorized statements on level two and three in the four-level hierarchical classification we used

	Total reviews	Total original papers	Threat (N original papers)				
			Direct effects	Habitat loss	Catastrophes	Pests and invasive species	Other indirect effects
1. Habitats to focus conservation measures on							
1.1 Which habitats to protect	**46**	**134**	**105**	**10**	**9**	**5**	**4**
Protect areas with a large range of habitats or high environmental heterogeneity	9	17	17	0	0	0	0
Protect currently intact environments	5	10	4	0	2	3	1
Protect forest habitats with specific values	11	29	27	0	0	0	2
Protect full range of bioclimatic variation	2	7	7	0	0	0	0
Protect habitats that can act as climatic refugia	26	50	44	7	4	0	0
Protect high quality habitats with a high homogeneity	1	3	3	0	0	0	0
Protect mountains	1	1	1	0	0	0	0
Protect transition zones between habitats	1	1	1	1	0	1	0
Protect wetlands, riparian zones, and coastal areas	7	14	13	3	0	1	0
2. Spatial distribution and size of protected areas							
2.1 Size of protected (or other kind of high-quality) areas	**31**	**82**	**67**	**14**	**6**	**4**	**5**
Protect a diversity of sizes of high quality areas within a network	1	2	2	2	0	0	0
Protect a few large high-quality areas	20	45	35	5	2	4	9
Protect a larger area in total	9	27	27	4	0	0	0
Protect intermediate amount of large enough areas	2	7	2	1	4	0	0
Protect many small high-quality areas	4	8	8	0	0	0	1
2.2 Spatial configuration, and patch connectivity of high-quality patches	**31**	**106**	**96**	**20**	**4**	**1**	**3**
Concentrate conservation measures	5	24	24	3	0	0	0
Decrease connectivity among protected areas	1	2	2	0	0	0	0
Ensure that back-up reserves are available	6	20	17	0	3	0	0
Increase connectivity between high-quality areas	14	33	32	4	1	0	0
Maintain corridors or stepping stones	25	56	46	16	0	2	0
Maintain networks of stopover sites for migratory species	3	3	3	0	0	0	0
Protect long areas	1	2	2	0	0	0	0
Protect round areas	1	1	0	0	1	0	0
2.3 Regional distribution	**17**	**40**	**38**	**5**	**0**	**2**	**3**
Ensure variation in management and areas protected	3	3	2	0	0	0	1
Protect areas based on future climate	13	32	31	3	0	0	2
Protect areas based on irreplaceability	2	4	4	1	0	1	0
Protect areas based on projected future range of species	1	1	1	0	0	0	0
Protect areas with a high evolutionary potential	2	2	2	1	0	1	0
2.4 Related to land-use intensity	**3**	**10**	**7**	**9**	**0**	**0**	**0**
Focus conservation efforts in human-dominated landscapes	3	10	7	9	0	0	0
3. Temporal aspects of protection							
3.1 Involve temporal aspects of protection	**8**	**13**	**12**	**2**	**0**	**0**	**2**
Consider seasonal aspects and succession when selecting area to protect	2	2	2	2	0	0	0
Protect land temporarily	6	10	10	0	0	0	2
4. Conservation measures related with land use and management							
4.1 Manage high value areas to reduce stressors	**4**	**16**	**16**	**0**	**0**	**0**	**0**
Identify and reduce stressors in and around Natural World Heritages	1	2	2	0	0	0	0
Identify and reduce stressors in and around protected forests	1	11	11	0	0	0	0
Mitigate other stressors than climate change in and around protected areas	2	5	5	0	0	0	0
4.2 Manage high value areas to increase resilience	**2**	**4**	**4**	**0**	**0**	**0**	**0**
Manage climate change refugia	2	4	4	0	0	0	0

## Results

We focus on those five categories of recommendations mentioned most frequently, with at least 5 review papers as a cut-off, and below we mention the most frequent recommendations first. Three types of recommendations occurred far more frequently than the others (*p* < 0.05, *χ*^2^ test comparing the value for the third and forth most frequent types of recommendations; Table 2). First, many recommendations regarded the spatial configuration of protected areas (2.2). Among these, a high proportion of papers highlighted that connectivity should be promoted to enable species to shift their distributions in response to the climate change or to recolonize patches in habitat networks. Second, many recommendations were related to the types of habitat to protect (1.1). These often highlighted that protected areas could act as climate refugia, and the recommendations thus were to protect certain habitat types that are particularly important as climatic refugia or to protect high habitat heterogeneity, and thus a high probability of capturing variability in micro-climatic conditions. Third, recommendations were frequently related to the size of protected areas (2.1). Most of these recommended that a few large areas should be protected, rather than several small. Fourth, multiple sources also recommended that protected areas should be located in areas that are predicted to be provide the most suitable environmental conditions as determined by projected climate change (2.3). Finally, it was recommended that land should be protected temporarily as a response to species’ distributions and shifting habitat suitability (3.1).

Approximately, 81% of the references used as support for recommendations in the reviews primarily addressed responses to the direct effects of climate change. The remaining 19% responded to various indirect effects: approximately 11% of papers made their recommendations to counter habitat loss, 3% invasive species, and 3% countered an increasing frequency of catastrophic events. All categories of recommendations, except one (‘focus conservation efforts in human-dominated landscapes’), were more frequently suggested as a response to direct rather than indirect effects of climate change.

In terms of which aspects of biodiversity were addressed, the references cited primarily considered biodiversity in general (66%), followed by a specific focus on certain species groups (21%), or habitats (7%), whereas genetic aspects (3%) and certain threatened species (2%) were only rarely considered. Ecosystem services were the main focus only for 1% of the references. Among these categories, there were only small differences regarding which categories of recommendations they were associated with; however, references focusing on threatened species tend to recommend protecting those habitats that act as refugia.

## Discussion

We found that numerous recommendations on how to adapt biodiversity conservation in protected areas to climate change have been published during the last decades, but these recommendations are not always consistent with each other. We suggest that these inconsistencies likely arose due to authors focusing on different underlying ecological processes. All original papers supporting recommendations considered preservation of biodiversity (and most often biodiversity in general) and only a few also considered ecosystem services. One explanation for this is that ecosystem services are less prioritized for consideration when addressing conservation strategies involving protected areas. In contrast, in an unpublished parallel review on conservation strategies in managed landscapes (Hämäläinen et al. subm ms.), the proportion of original papers that supported recommendations aimed to benefit ecosystem services was considerably higher (25%).

### Spatial configuration of habitat: Increase landscape connectivity

The spatial configuration of protected areas was often emphasized in the reviews, especially the need for increased connectivity. This is consistent with classic principles in conservation biology (e.g. Diamond 1975), upon which climate change appears to provide an added impetus to facilitate dispersal (e.g. Opdam and Wascher 2004; Fourcade et al. 2021). Thus, this category of recommendations means that additional efforts should be made enhancing already existing conservation strategies.

**Fig. 3:**
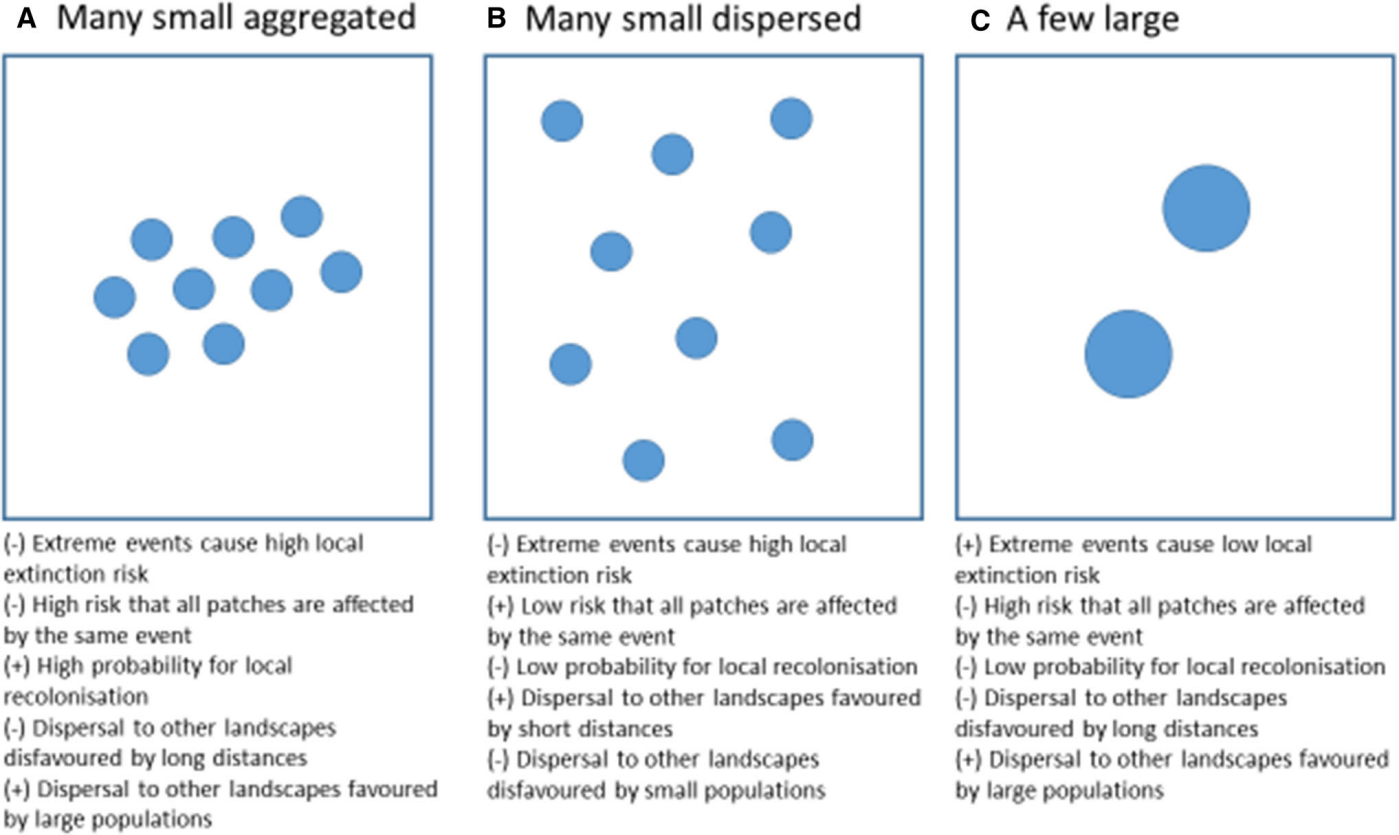
Spatial distribution of protected areas, resulting from three different strategies that might be modified as a response to climate change. **A** Generates a large risk for local extinctions but also a high patch connectivity. Consequently, if a species goes extinct from one site there is a high probability that this site will be recolonized by migration from other sites. However, the long distance from this cluster of sites to other possible clusters makes dispersal through the landscape more difficult. **B** Has a lower patch connectivity in comparison to **A** which makes recolonization less likely and decreases landscape-level population size. When local extinctions have causes that are spatially correlated, **B** generates a lower risk that many patches are affected at the same time, which decreases the overall extinction risk. Short distances between patches in other landscapes facilitate dispersal through the landscape. **C** Has the lowest probability of local extinction, since the populations are larger and a larger area can buffer for many disturbances. The probability for dispersal tends to increase due to large populations and decrease due to long distances to other patches. Which of these strategies are the best depends on the extent and characteristics of the disturbances, and the need for and capacity of the landscape to allow for species persistence and dispersal through the landscape, which in turn is affected by the biology (e.g., dispersal ability) and habitat requirements of species

In the scientific literature, there is a distinction with respect to the specific meaning of “connectivity” (Fahrig et al. 2021). In metapopulation ecology, “patch connectivity” (or “Hanski connectivity” sensu Fahrig et al. 2021) is a characteristic of a habitat patch reflecting the potential for immigration. This potential increases with the number and size of populations in surrounding landscape and decreases with their distance (e.g., Hanski 1994). This differs from “landscape connectivity” (or “Merriam connectivity” sensu Fahrig et al. 2021), which is a characteristic of landscapes reflecting the degree to which they facilitate migration (Tischendorf and Fahrig 2000). Patch connectivity is important for species persistence in fragmented landscapes independent of climate change. However, it is expected to become even more important in a changing climate; because local extinctions tend to become more frequent with a higher frequency of extreme climatic events. Thus, there is a need for more immigrants contributing to recolonizations (Fig. 3A; Timpane-Padgham et al. 2017). Climate change also tends to make landscape connectivity more important. This is because the resultant changes in environmental conditions cause species to shift their potential distribution ranges, and as a result, it may be necessary for species to migrate through less suitable areas to reach regions with improved conditions (Fig. 3B; Fourcade et al. 2021). Higher landscape connectivity makes successful migration through such areas more likely. Thus, improving patch connectivity targets the viability of current in situ populations despite the stress of climate change, whereas improving landscape connectivity targets the capacity of species to address climate change via adjusting their spatial distribution.

Both patch and landscape connectivity grow with an increased amount of habitat, permeability of the matrix, creation of corridors between high-quality areas, and decreased contrast between reserves and their surroundings (Samways 2007). However, for a metapopulation to persist, to increase patch connectivity by aggregating habitat in a certain part of a landscape (where the density of patches generate enough recolonizations; Fig. 3A), is better than a more even distribution of habitat (Fig. 3B) (Hanski 2011). On the other hand, a more even distribution throughout the landscape may be more efficient at promoting landscape connectivity (Keeley et al. 2018), facilitating the ability of species to shift their distributions (Fourcade et al. 2021). Another argument for spatially dispersed reserves is the increased risk for catastrophes caused by a warming climate, since longer distances between reserves minimizes the risk that they are all affected by the same catastrophe (Schafer 2001). Corridors may also need to be differently designed; for patch connectivity, corridors between any patches are valuable, while climate change makes it more important to ensure connection between warmer and cooler sites (Keeley et al. 2018).

To sum up, patch connectivity will remain important, but climate change increases the importance of facilitating long-distance movements between landscapes (Opdam and Wascher 2004). How to prioritize between these two aspects depends on the relationship between a landscapes’ capacity to preserve biodiversity (often the need for patch connectivity decreases when the amount of remaining natural habitat increases; Andrén 1994) and the vulnerability of habitats to climate change (a higher vulnerability increases the need for facilitation of long-distance movements) (Gillson et al. 2013).

### Type of habitat to protect: Focus on climate refugia

Many studies recommended that when protecting areas, certain habitat types should be prioritized. In particular, protected areas encompassing a diverse range of micro-climates were recommended for prioritization, due to the expectation that these could act as refugia for species. This consideration has developed as a consequence of climate change. Habitats such as wetlands, riparian zones, and forests were also recommended for prioritization (Junk et al. 2013; Kuuluvainen and Gauthier 2018). The recommendations to focus on these habitats are consistent with conservation strategies developed in the absence of climate change.

A climate refugium can be defined as an area with a low climate velocity, i.e. with a slow movement of isotherms (= contour lines along which the temperature is equal) over time during climate warming (Keeley et al. 2018). Within a climate refugium, the expectation is that species can find suitable climate inside a limited area over extended time periods, despite changes in the regional climate. The more the climate changes, the higher proportion of climate refugia eventually become too warm. However, even when species cannot persist in the long term, climate refugia can be important as stepping stones, since they act as dispersal sources over some time, increasing the probability for colonisation of sites that are more suitable in the longer term (Hannah et al. 2014; Morelli et al. 2020). At larger scales, climate refugia can be defined as an area where the current and the predicted future distribution areas of species overlap (Jones et al. 2016). During the last two decades, much efforts have been spent on predicting potential future distribution areas by combining knowledge about climatic conditions (recently also microclimate: Stark and Fridley 2022) where species occur today with predictions about future climate (Heikkinen et al. 2006; Rasmont et al. 2015). However, for most species, lack of detailed knowledge of species distributions, as well as difficulties in selecting predictors that reflect species’ actual niches (Fourcade et al. 2018) make such predictions difficult or unreliable (Lobo 2016). At smaller spatial scales, climate refugia can be identified by utilizing high-resolution climate data (Greiser et al. 2018), but such data is often lacking (Keeley et al. 2018). However, recommendations to preserve climate refugia can be formulated more generally, and thereby bypass the need for high resolution climate data, by describing the landscape components to focus on, such as valleys, forests, north-facing slopes, and sites close to large water bodies (Morelli et al. 2016; Greiser et al. 2018), as well as ecotones and other areas with high levels of environmental heterogeneity (Lawler 2009). Such recommendations can be followed anywhere these landscape features exist, while still acknowledging the benefits of obtaining more context-specific data to improve the precision of such recommendations.

In the literature, particular emphasis was placed on protecting riparian zones. One reason for this is that the risk for flooding and drought events is projected to increase in some regions, and protecting riparian zones may increase resistance to these disturbances (Timpane-Padgham et al. 2017). This can also be true for other types of wetlands. Riparian zones can also act as climate refugia, since they often have steep gradients in vegetation cover and moisture conditions. Furthermore, riparian zones might act as dispersal corridors, facilitating species movements through landscapes, and if so they also contribute to landscape connectivity (Keeley et al. 2018).

Arguments were also made to protect habitats with long temporal continuity, such as old forest (Noss 2006). One reason for this is that climate change might lead to an intensified land use regime, including a decrease in the availability of unmanaged late-successional forests and shorter rotation periods in production forests (Felton et al. 2016). Old forest harbours structures important for biodiversity that are lacking in younger forests, and thus climate change indirectly increases the urgency of preserving such forest (Kuuluvainen and Gauthier 2018). More directly, climate change tends to increase habitat turn-over due to more frequent weather-related disturbances. Theoretical analyses show that increased habitat turnover directly contributes to increased metapopulation extinction risk (Keymer et al. 2000), which is also a  reason why it is important to increase the proportion of terrestrial areas subject to low rates of turnover (van Teeffelen et al. 2012). Conclusively, projected changes of both disturbance regimes and land-use provide added emphasis to protecting old forest and other habitats with a long temporal continuity.

### Size of protected areas: Protect a few large

Recommendations were often related to the size of protected areas. A majority of these recommendations suggested that a few large areas should be protected (Fig. 3C), even though arguments for many small reserves were also found (Fig. 3A and B). Moreover, it was often stated that with climate warming, the total protected area has to increase if specific conservation goals are to be reached. These recommendations do not imply any new approaches to biodiversity conservation, but provide added emphasis to well established conservation strategies.

In conservation biology, it has long been debated whether it is better to preserve a few large or many small areas (Diamond 1975). This dilemma has often been referred to as the SLOSS debate, as an acronym for Single Large Or Several Small. Consequently, there are a range of established supportive and detracting positions associated with either alternative. A main argument for a few large protected areas is that it results in a lower long-term extinction risk for species with restricted ability to move between areas. On the other hand, since species composition typically differs among sites within a landscape, many small protected areas can potentially cover a larger variety of species communities located throughout a landscape, and thus help conserve more biodiversity (Fahrig et al. 2022). Since it is difficult to test under which conditions a few large or many small areas generates the best outcome overall, such studies are still lacking (Fahrig et al. 2022). Climate change provides its own specific context from which to consider these arguments anew (Fig. 2). For example, larger reserves harbour, on average, a larger variety of environmental conditions. Therefore, species may be better able to persist in such reserves as the conditions provided may enable them to disperse to sites within the reserve providing suitable conditions for them in the future (Lawler 2009). However, for species that can disperse between patches, many small reserves can be more favourable since they have the potential to cover a wider gradient of both current and future microclimates occurring in the landscape (Carroll and Noss 2021). Moreover, climate change tends to increase the frequency of disturbances, and that will generally increase local extinction risks. To increase the size of the reserves constitutes a way to compensate for these losses (van Teeffelen et al. 2012). However, the opposite conclusion could also be drawn: with more frequent disturbances it is more important to spread the risk by preserving many small areas (Fahrig et al. 2022). Other arguments for the use of large protected areas are more habitat specific. For instance, in forest dominated protected areas, reserves that are larger or surrounded by buffer zones may be more resistant to the consequences of climate change than smaller ones (Noss 2006). Another example involves coastal areas, where large reserves with an extension from the sea to the inland may allow the persistence of coastal species when sea levels rise (Lawler 2009). In contrast, an advantage with many small reserves is that they can act as stepping stones (Hodgson et al. 2012), thus increasing landscape connectivity (see *Spatial configuration of habitat*). Thus, as per the concluding sentiments of the SLOSS debate of the past, in the future there is also likely to be a need for both large protected areas (for preserving the most area-demanding habitats and species) and a greater number of smaller reserves (to spread the risk and to cover the variation in habitats, climate, and biodiversity within landscapes).

It is commonly argued that the total area protected for biodiversity conservation needs to increase due to climate change. One reason for this is that climate change increases the risk that reserves are destroyed by catastrophic events (Schafer 2001). Even in the absence of single devastating events, less stable environmental conditions induced by climate change are likewise generally considered negative (van Teeffelen et al. 2012). Thus, to preserve the regional species requires more reserves than if the conditions were to remain more stable.

### Regional distribution: Consider conservation values in a future climate

Several reviews recommended that future climatic conditions should be considered when selecting which areas to protect. This is a strategy strictly associated with climate change, and requires new approaches and knowledge to be enacted.

Global warming is causing a shift in the range of species and habitats to relatively cooler areas located at higher altitudes and latitudes (Lenoir and Svenning 2015). As a result, protected areas located at higher altitudes and towards the poles are expected to increase in value as lower altitude and latitude species enrich these areas in the future, and likewise, because some of the species dependent on current environments found in these sites, are expected to become more threatened in the future (Li et al. 2006). Considering these changes, areas prioritized for protection can be identified by utilizing climate scenarios and biological data that predict the future distribution of species (Jones et al. 2016). By doing so, it is possible to consider the level of ‘irreplaceability’, i.e. the potential contribution of a reserve to a conservation goal, while taking into account the rarity of its future habitats (Samways 2007) or climate (Ohlemüller et al. 2008). In the absence of such data intensive modelling efforts, a more general rule can be applied, namely that protected areas located in cooler regions will become more valuable for biodiversity conservation in the future and these regions are typically situated closer to the poles or at higher altitudes.

### Temporal aspects: Protect moveable reserves

Some reviews recommended that in addition to protecting areas permanently, there should also be temporarily protected areas, known as moveable reserves or dynamic protected areas (Reside et al. 2018). Contracts for temporary protection are already used on privately owned land. This can increase landowner’s acceptance for area protection, but seems to be a cost-efficient way to protect biodiversity only in the short term (Mönkkönen et al. 2011). This protection strategy has been questioned (Moilanen et al. 2014), but may become more relevant as a targeted response to the consequences of climate change.

The fact that climate change is expected to make habitats less stable can be considered in biodiversity conservation both with respect to mitigating its negative effects and in terms of adaptation. One recommended mitigation measure is to protect more areas with a long habitat continuity, such as old forests, since they tend to become rarer (see *Type of habitat to protect*). Thus, this strategy targets late-successional habitats. On the contrary, one recommended adaptation measure is to protect some areas only with short-term contracts, which mainly targets early-successional habitats. This can be useful, for instance, when disturbances have made certain sites (for instance, fire or drought refugia) particularly important for biodiversity during a limited period of time (Reside et al. 2018). Such areas can also track predicted shifts in species’ ranges, and thus facilitate colonisation by these species (Alagador et al. 2014). However, the risks associated with temporary protection can be considerable, both in terms of environmental and financial uncertainties (Moilanen et al. 2014). Especially with respect to late-successional habitats, moveable reserves are less attractive, since large investments in conservation is lost at the end of each contract period.

## Conclusions

We focused on the five most frequently mentioned types of recommendations (Fig. 1), even though in the literature, a wide range of additional conservation measures was also recommended (Table 2). Some of the most recommended conservation measures involve an increased application of the same principles that have influenced nature conservation prior to concerns being raised about climate change (e.g., increase patch connectivity, protect large areas, protect certain habitats). In addition, climate change impacts have catalyzed the need for modified and new principles to be formulated (e.g. increase landscape connectivity, protect climate refugia, consider conservation values in a future climate, and protect moveable reserves). Among those recommendations motivated by the indirect effects of climate change, the continued application of old ecological principles often dominated, because these indirect effects are the most similar to the existing threats caused by land use. The recommendations motivated by the direct effects of climate change were based on both old and new ecological principles. As conservation funding is limited, prioritization among these recommendations is unavoidable. The most cost-efficient combination of conservation measures should be applied, but in the literature we found no examples of any formal analysis of the cost-efficiency of adapting conservation measures to climate change.

Given the recommendations we found, there are inherent trade-offs to be addressed; for instance, increased permanent protection of a large high-quality areas implies that less resources remain for the creation of corridors, stepping stones, or temporary reserves -  all of which also can find support within the scientific literature. Which of these conservation strategies should be prioritized in turn depends on future conservation goals, characteristics of future landscapes, and inhabiting species communities, for which there are large uncertainties. One way to handle uncertainties regarding how the climate and society will develop is by considering several different contrasting futures, and applying strategies with projected benefits even under a wide range of potential futures (Jones et al. 2016).

Uncertainties stemming from our ecological knowledge are also important. For some principles formulated decades ago, there are still large uncertainties that can stem from the lack of studies over large spatial or temporal scales, or that the observed patterns may be context-specific and thus of limited general applicability (e.g., for SLOSS: Fahrig et al. 2022). Novel approaches that intend to adapt biodiversity conservation to a warming climate are even more difficult to evaluate, and thus, for these approaches the uncertainties are even larger. Therefore, extensive monitoring is needed to evaluate both the need for different adaptation strategies to climate change, and the effectiveness of the various strategies that are applied (Morecroft et al. 2019). Unfortunately, often before such evaluations take place, decisions will have to be made regardless of these large uncertainties.

In general, climate change implies that more conservation efforts are needed to achieve conservation goals (cf. Stein et al. 2013). This enhanced need for protected areas stems from both the additional stressors induced by climate change and the inherent uncertainties involved in projecting its impacts, and the associated response of species. For instance, in the absence of climate change a particular species community might be effectively preserved in a certain network of protected areas. With climate change, this network needs to be larger, because more extreme weather conditions readily increase the risk of local extinction. Furthermore, it is important to ensure that this network includes climate refugia with conditions suitable for species over a longer time, and also that the network is connected to other regions that are projected to have a more suitable climate in the future. Note, however, that in regions where many species are at their coldest parts of their distributional range, sites with the warmest microclimate are expected to be the most species rich. To preserve biodiversity in such regions might become easier with climate change (Müller et al. 2015).

## Supplementary Information

Below is the link to the electronic supplementary material.

Supplementary file1 (PDF 197 KB)

Supplementary file2 (XLSX 147 KB)
