# Supplementary material for: Protected area designation and management in a world of climate change: A review of recommendations
Source: Ambio. 2022 Aug 23;52(1):68–80. doi: 10.1007/s13280-022-01779-z (PMC9666604; doi:10.1007/s13280-022-01779-z)
Supplement: Supplementary file 1 — Supplementary file1 (PDF 197 KB) [file 13280_2022_1779_MOESM1_ESM.pdf]

Ambio

Supplementary Information

This Supplementary Information has not been peer reviewed.

Title: **Protected area designation and management in a world of climate change: A review of recommendations**

Appendix S1. Search strings used when searching for literature

We combined the three first search strings (included in all searches) with five other search strings (included one at a time). This paper considers recommendations for protecting areas or conservation management in protected areas. The search described below however, also included recommendations for promoting biodiversity by adapted management in forestry and agriculture, which are summarized in a paper by Hämäläinen et al. (subm.ms), and were thus excluded from the present study. Below is given the exact search strings used in Web of Science, and equivalent versions were used in Scopus.

Search strings included in all searches, with AND in between these strings

Management: TS=(adapt* OR manag* OR compensat* OR restor* OR offset* OR strateg* OR planning* OR land-use* OR ”land use*” OR “protected area*” OR reserve* OR “national park*”)

Goal: TS = (conservat* OR biodivers* OR “species diversity” OR “red list*” OR “threaten*” OR “endanger*”)

Other refining arguments: LANGUAGE: (English) AND DOCUMENT TYPES: (Review)

Timespan: 1998-2018.

Effects of climate change (included one at a time)

1) Changed climatic conditions

temperature, precipitation, snow

TS = (“climat* change*“ OR “global warming“ OR “changing climate“ OR “increased temperature*“ OR “temperature rise“ OR “temperature* rising“ OR “raised temperature*“ OR “higher temperature*“ OR “increase* precipitation*“ OR “precipitation* rise“ OR “precipitation* rising“ OR “raised precipitation*“ OR “higher precipitation*“ OR “lower* precipitation*“ OR “decreased precipitation“ OR “precipitation decline“ OR ((increas* OR decreas* OR chang* OR shift* OR alter*) AND (”snow cover*” OR “snow depth*” OR “snow period*”)))

2) Disturbances and catastrophes

fire, storms, flooding, drought, sea level change

TS =(((chang* OR shift* OR alter* OR increas*) AND (fire* OR wildfire* OR “storm felling*“ OR “sea level*“ OR “coast line*“ OR flood* OR “shore line*“ OR “sea shore*“ OR drough*)) OR (calamity AND forest*))

3) Habitat loss or changes

vegetation (incl. tree species) composition, area of forest and agricultural land

TS = (((change* OR shift* OR altered*) AND (“tree species composition*“ OR “tree composition*“ OR “vegetation composition*“ OR “vegetation type*“ OR “forest type*“ OR “habitat type*“)) OR (increase* AND (broadleaved OR “deciduous tree*“ OR "deciduous forest*")) OR (“habitat loss*“ AND (forest* OR woodland*)) OR (“habitat loss*“ AND (agriculture* OR grassland* OR pasture* OR meadow* OR farmland*)))

4) Length of vegetation season and forestry rotation

rotation length in forestry, vegetation season in agricultural land, grazing period

TS=((shorte* OR extend* OR lengthen* OR modifie* OR chang* OR alter* OR shift*) AND (“rotation length*“ OR “rotation time*“ OR “rotation period*“ OR “rotation cycle*“ OR “harvest* age“ OR “harvest* time“ OR “cutting age“ OR “harvest* cycle*“ OR “vegetation period*“ OR “grazing period*“ OR “vegetation season*“ OR “growing season*“ OR “growing period*“))

5) Pests and invasive species

invasive species, pests and pathogens, and their control

TS=(((exotic* OR invasive* OR alien* OR “non-native*”) AND (species OR biol* OR ecol*)) OR ((increase* OR higher*) AND (pest* OR pathogen* OR decease*) OR (pesticide* OR insecticide* OR fungicide* OR herbicide* OR weedicide* OR “weed control*”)))

Appendix S2. Final version of inclusion criteria.

1. The study should address conservation efforts in response to or as a precaution to any of the effects of climate change given in Table 1. Any direct or indirect effects of climate change should be mentioned, or it should be clear that they compare management that works in different climatic conditions, or for different levels of any of the indirect effects.
2. The aim of the conservation efforts should be to preserve biodiversity (diversity of species, genes or habitats), certain threatened species, or ecosystem services (ES) dependent on biodiversity. Included ES were pollination, seed dispersal, pest control (including invasive species), disease control and soil quality in terms of decomposition and fixing processes (CICES v5.1). Biodiversity could also include single cell organisms.
3. The study should conduct a review of the field, summarizing or compiling the different management suggestions that exists for that topic and indicate papers that evaluate these. Meta-analysis and narrative syntheses that compile previous studies are included, while pure modelling studies were not.
4. The findings should be applicable to terrestrial and limnic habitats in a wide range of forest and agricultural landscapes in Northern Europe or Northern America. This resulted in our inclusion of
   i) studies from the Northern hemisphere within climate zones Dfb, Dfc, Cfb and Cfc according to the Köppen-Geiger climate classification (Kottek *et al.* 2006).
   ii) reviews covering several climate zones if a large part of the studies covered by the review were from the abovementioned zones. If the vast majority were from other conditions they were not included. Recommendations clearly supported only for other zones were excluded.
   iii) global reviews.
   iv) cities, if it is possible that forests or agricultural land was included.
   v) mountain areas, if it was not clear that forest and agricultural land was absent.
5. We included conservation measures that are active and on-ground actions taken to minimize negative impact.

6. We excluded theoretical frameworks that only identify the circumstances within which the actions should take place, e.g., “adaptive management” or a new “framework”.
7. The conservation efforts should be related to area protection for nature conservation or take place at protected areas.
